# Targeting metabolic/epigenetic pathways: a potential strategy for cancer therapy in diffuse intrinsic pontine gliomas

**DOI:** 10.1038/s41392-020-00344-y

**Published:** 2020-10-06

**Authors:** Kun Zhao, Hongming Miao

**Affiliations:** Department of Biochemistry and Molecular Biology, Army Medical University, 400038 Chongqing, China

**Keywords:** Cancer metabolism, Cancer therapy, CNS cancer

A recent study published in *Cancer Cell* by Chan Chung and colleagues demonstrated that H3.3K27M mutation in diffuse intrinsic pontine gliomas (DIPGs) potentiated glycolysis, tricarboxylic acid cycle, and glutaminolysis metabolism with upregulated alpha-ketoglutarate (α-KG), which contributed to sustain an epigenetic status marked by H3K27me3 deficiency and that interruption of these metabolic/epigenetic pathways represented a promising strategy for the treatment of DIPGs, as reprted by Chung, C. et al.^1^

**Fig. 1:**
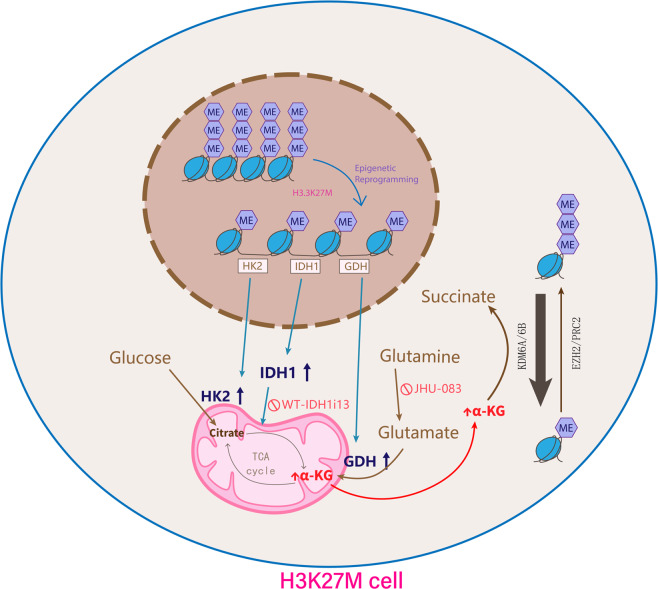
Schematic model of interplay between metabolic and epigenetic pathways in H3K27M cells. Elevated α-KG from enhanced glycolysis, glutaminolysis and tricarboxylic acid cycle metabolism inhibits H3K27me3 levels in H3.3K27M mutant cells. Reduction in H3K27me3 levels promotes gene expression including *Hk2*, *Idh1*, and *Glud1*, which enhances glycolysis, tricarboxylic acid cycle and glutaminolysis metabolism along with increased production of α-KG, which functioned as a vital cofactor of KDM6A/6B, resulting in demethylation of H3K27. As a result, α-KG was metabolized to succinate while demethylating H3K27me3. Noteworthily, two pharmaceuticals JHU-083 (a glutamine antagonist) and WT-IDH1i13 (an *IDH1* inhibitor) were therapeutic for DIPGs. Glud1 glutamate dehydrogenase 1, GDH glutamate dehydrogenase, Hk2 hexokinase 2, Idh1 isocitrate-dehydrogenase 1, α-KG alpha-ketoglutarate

H3K27M, which refers to that the methionine replaces lysine at site 27 in histone H3-*H3F3A* and *HIST1H3B/C* (collectively H3K27M),^1^ takes place in more than 80% of diffuse intrinsic pontine gliomas (DIPGs).^2^ H3K27M mutations account for a general H3K27me3 deficiency through varied mechanisms, such as its aberrant interactions with PRC2.^3^ It is reported that epigenetic modification can increase global H3K27me3, which has particularly shed light on a novel therapy of gliomas by inducing H3K27M cell death.^4^ However, the relationship between metabolic alterations and epigenetic modifications in H3.3K27M DIPGs are still not fully understood. In the present study, Chung et al. discovered that there was potentiated glycolysis, tricarboxylic acid cycle, and glutaminolysis metabolism in H3K27M cells along with a high level of alpha-ketoglutarate (α-KG). They fully proved that there existed an interplay between metabolic and epigenetic pathways in H3K27M tumors (Fig. 1).

To determine the alterations in metabolism driven by H3.3K27M mutations, the authors made use of cell lines with H3.3K27M mutation, such as immortalized mouse neuronal stem cells (H3.3K27M NSCs), patient-derived tumor cell lines (DIPG-IV, DIPG-XIII*p, DIPG-007, and SF7761), and tumor samples. All integrated approaches including RNA sequencing, proteomics, and metabolomics in H3K27M NSCs comprehensively demonstrated that these cells displayed augmented glycolysis and TCA cycle, which were mediated by activated factors like *GLUT3*, *GLUD1* (encoding GDH), *HK2*, and *IDH1* confirmed by chromatin immunoprecipitation sequencing (ChIP-seq). Homologous results were verified in cells from patient-derived tumors by means of single-cell (sc) RNA-seq analysis. What’s more, enhanced glutaminolysis was also certificated by isotope tracing, which paralleled with the magnetic resonance spectroscopy (MRS) imaging in samples of patients suffering from high-grade midline gliomas.

Since the metabolic changes mediated by H3.3K27M mutations are fully clear, it is important to study the relationship between H3.3K27M, glycometabolism and glutamine metabolism. α-KG, which was turned out to be upregulated in H3.3K27M cells through metabolomics, is a crucial cofactor of H3K27 demethylases KDM6A/6B.^5^ α-KG is metabolized to succinate (Suc) by KDM6A/6B and leads to H3K27me3 demethylation. Based on that H3.3K27M cells showed high α-KG levels but low Suc contents, Chung et al. looked forward to proving that H3K27me3 levels could be modulated by α-KG in these cells. Authors found that glutamine or glucose deprivation from cell culture medium could increase the H3K27me3 contents in patient-derived cells (e.g., DIPG-007 and DIPG-IV), and this effect could be abolished by affixion of α-KG, which can be assimilated into cells. At last, the authors made a conclusion that α-KG was important for holding an epigenetic status marked by H3K27me3 deficiency in H3.3K27M cells.

In addition, authors also determined whether overall H3K27me3 levels and cell proliferation could be influenced by impeding glutamine or glucose metabolism in H3K27M cells. Results showed that genetic (e.g., shRNAs) or pharmacological interruption (e.g., enzyme inhibitors) of *GDH*, *HK2*, or *IDH1* obviously lowered α-KG/Suc ratios, increased H3K27me3 levels and suppressed H3.3K27M cell growth in vitro and in vivo. Surprisingly, ATAC-seq analysis demonstrated that *GDH*, *HK2*, or *IDH1* silence in DIPG-007 cells contributed to increase chromatin accessibility, which were associated with genes about neuroglial differentiation, indicating an abrogation of malignant progression. Next, the authors found two pharmaceuticals, JHU-083 (glutamine antagonist DON analog) and WT-IDH1i13 (micro molecules covalently suppress WT-IDH1), both of which have high BBB penetrability. Results showed that maximal therapeutic effect in prolonging overall survival in DIPG mouse models was observed on combined treatment with JHU-083 and WT-IDH1i13, indicating that combined suppression of α-KG-encoding enzymes in glucose and glutamine metabolism had a synergistic enhanced therapeutic effect on DIPG mouse models, which were established by transplanting DIPG-XIII*p or DIPG-007 cells into the pons.

Taken together, the authors revealed that tumors with H3.3K27M mutant could promote both glutamine and glucose metabolism for α-KG production to sustain their growth and made it clear that there existed a strong interplay between metabolic and epigenetic pathways in H3.3K27M cells. Those findings shed light on the therapy of DIPGs by targeting metabolic/epigenetic pathways.
